# Human Computer Interface for Tracking Eye Movements Improves Assessment and Diagnosis of Patients With Acquired Brain Injuries

**DOI:** 10.3389/fneur.2019.00006

**Published:** 2019-01-23

**Authors:** Michał Lech, Michał T. Kucewicz, Andrzej Czyżewski

**Affiliations:** ^1^Multimedia Systems Department, Faculty of Electronics, Telecommunication and Informatics, Gdansk University of Technology, Gdańsk, Poland; ^2^Mayo Clinic, Department of Neurology, Rochester, MN, United States

**Keywords:** consciousness level assessment, gaze tracking, eye movements, awareness, Cyber Eye

## Abstract

One of the first clinical signs differentiating the minimally conscious state from the vegetative state is the presence of smooth pursuit eye movements occurring in direct response to moving salient stimuli. Glasgow Coma Scale (GCS) is one of the most commonly used diagnostic tools for acute phase assessment of the level of consciousness, together with a neurological examination. These classic measures are limited to qualitative neurological examination without more quantitative measures provided from e.g., tasks with tracking position of the gaze. Among this and other limitations, it is prone to a relatively high rate of misdiagnosis. Here, we developed an interface for gaze tracking to enhance the assessment of consciousness in 10 patients with acquired brain injuries. According to the acute phase GCS assessment, nine of them were considered unaware and below the minimally conscious state. Chronic neurological examination confirmed six of them below the minimally conscious state. Our new Human Computer Interface (HCI) revealed that six patients were conscious enough to complete at least one of the gaze tracking tasks. Among these six patients, one was originally diagnosed as remaining in a vegetative state and one in coma. The patient diagnosed as remaining in a chronic vegetative state scored six GCS points acutely. Following assessment with our HCI the patient was re-diagnosed with a possible locked-in syndrome. Our HCI method provides a new complementary tool for clinical assessment of patients suffering from disorders of consciousness.

## Introduction

Glasgow Coma Scale (GCS) is the most commonly used method for assessing consciousness level in the acute phase after acquired brain injuries. This acute phase assessment is typically followed by repeated neurological examination in the chronic phase. Alternative consciousness scales have been proposed but none has currently gained a comparable popularity (1, 2). Several shortcomings of the acute phase GCS and chronic neurological examinations have been reported for the last two decades (3–6). One of the main drawbacks is that the eye movements are not being tracked in the these assessments. The presence of smooth pursuit eye movements in response to a moving or salient stimulus is a critical clinical sign differentiating the minimally conscious state from the vegetative state (6). Several alternative scales use various stimuli to evaluate the visual smooth pursuit (6, 7). A moving mirror is used in the Coma Recovery Scale-Revised (CRS-R) (8) and in the Western Neuro-Sensory Stimulation Profile (WNSSP) (9), whereas a finger movement is used in the Coma/Near Coma Scale (10), the Wessex Head Injury Matrix (WHIM) (11), the Sensory Modalities Assessment and Rehabilitation Technique (SMART) (12), and in the Full Outline of UnResponsiveness scale (FOUR) (1, 13). This additional assessment of eye movements enables patients in minimally conscious state to be more accurately identified compared to the GCS assessment. Furthermore, the FOUR scale specifically tests for voluntary eye movements or blinking in response to a command to open eyes, enabling early detection of the locked-in syndrome. This is critical for diagnosing rare cases of the syndrome, which is not detected in up to 50% of the cases (4). During the validation of the FOUR scale (14), the scale enabled us to identify 11% of patients who presented non-verbal signs of consciousness and thus remained unidentified in the GCS assessment. Overall, despite the existence of multiple methods for assessing the consciousness level, erroneous diagnosis is reported in up to 43% of the cases (15).

The high error rates in the above-mentioned scales can be explained by a clinical need to provide a fast assessment of patients' state when early diagnosis is crucial. As such they were not designed to provide more in-depth information about cognitive processing in the brain. Moreover, it is inherently difficult to quantify their measures, for instance, when assessing eye movements in response to tracking position of a finger or a mirror. Eye-tracking technologies present a promising aid to make assessments of consciousness more precise, accurate, and effective (16).

To address these issues, we developed a Human Computer Interface (HCI) technology for a more accurate and quantifiable assessment of the consciousness level. Our proposed HCI is based on tracking eye movements to follow position of the gaze on a computer screen during performance of simple cognitive tasks. We hypothesized that consciousness level can be more accurately identified by examining patient's ability to complete the gaze-tracking tasks. The technology can also hypothetically provide a deeper insight into the underlying cognitive impairments, including aphasia, alexia, or agraphia.

The gaze tracking technology gains popularity in biomedical engineering both for diagnostic and communication purposes. It is employed in research of severe neurological disorders like sclerosis (17) and visuospatial neglect (18). Trojano et al. (19) employed a computerized infrared 60-Hz eye-tracker system in assessment of characteristics of visual tracking in 18 DoC (disorders of consciousness) patients and in 11 healthy control participants. Among the DoC patients nine were in vegetative state (VS) and nine were in minimally conscious state (MCS). The proportion of on- or off-target fixations differed significantly between VS and MCS patients. Moreover, “the distribution of fixations on or off the target in all VS patients was at or below the chance level, whereas in the MCS group seven out of nine patients showed a proportion of on-target fixations significantly higher than the chance level” (19). These findings motivate the need for creating the gaze tracking devices and attempts of employing them in clinical practice.

In our system the gaze tracking technology is used both for controlling the computer while performing the tasks and for diagnostic purposes through analysis of the registered gaze responses. The technical framework of our HCI has been previously reported (20, 21), revealing a weak correlation between the GCS scores and the eye-tracker signals. Here, we tested the clinical feasibility of the HCI in assessment of the level of consciousness of patients suffering from acquired brain injuries.

## Materials and Methods

The goal of the study was to assess the level of consciousness using objective measures of gaze tracking during two consecutive sessions with simple computer tasks. This new assessment was compared with the chronic neurological examination of the consciousness level. In addition, the acute phase GCS (the original 1974 version) assessment results have been presented in the paper to provide the information on initial state of patients. According to the original GCS, eye responses were assessed (4 point scale), together with verbal and motor responses (5 point scale). The neurological examination, in which no scale was applied, was performed by the clinician neurologist on the day of the incident and it was repeated annually. Although the purpose of the GCS is to assess patient's responses acutely, it is a common practice to use the scale for monitoring the responses chronically as well, as practiced in the medical center where the study was conducted. Both the basic neurological examination and the GCS assessment were additionally performed on the day of conducting the first session with each patient (Table 1). All patients showed consistent results of the neurological examinations over time, starting with the very first assessment performed on the day of their incident. The neurological examination (with no scale) as well as the diagnosis was made by a neurologist with long-standing experience with head trauma and acquired brain injuries. The GCS assessment was performed by a clinical therapist assisted by a physiotherapist in the motor response assessment. The verbal response was assessed solely by the clinical therapist, who specialized in aphasia, alexia and agraphia therapies. The eye response was assessed in cooperation of both the clinical therapist and the physiotherapist.

**Table 1:** Original clinical epicrisis of patients diagnosed based on standard neurological examination following the acute phase Glasgow Coma Scale assessment of consciousness state.

	**Type of incident**	**Date of incident**	**Date of conducting the first session**	**Age range**	**Diagnosis**	**GCS assessment**			
						**E**	**V**	**M**	**Sum**
P1	Cardiac arrest (heart attack, stroke)	2015-07	2017-12-14	55–60	Vegetative state	2	1	2	5
P2	Ischemic stroke	2016-10	2017-12-15	60–65	Vegetative state	4	1	3	8
P3	Cardiac arrest	2016-12	2017-12-14	35–40	Vegetative state	4	1	2	7
P4	Cardiac arrest (brain tumor)	2016	2017-12-14	55–60	Vegetative state	4	1	1	6
P5	Cardiac arrest (hemorrhagic stroke)	2015-02	2017-12-14	50–55	Minimally conscious state	4	2	4	10
P6	Cardiac arrest (alcohol poisoning)	2015-07	2017-12-21	30–35	Akinetic mutism, cortical brain and cerebellum atrophy	3	2	3	8
P7	Cranio-cerebral trauma (traffic accident)	2015-03	2017-12-22	45–50	Minimally conscious state	4	1	3	8
P8	Cardiac arrest (multifocal brain damage)	2017-06	2018-02-23	70–75	Minimally conscious state	4	1	3	8
P9	Cardiac arrest (suicide attempt)	2014-09	2018-03-09	20–25	Coma	3	1	3	7
P10	Traffic accident	2005-08	2018-03-08	30–35	Vegetative state	4	1	2	7

*E, eye response; V, verbal response; M, motor response*.

Written informed consent for participation was obtained from guardians of all participating subjects according to the Ethics Committee of approval of the clinic (more details included in Declarations). We recruited patients who satisfied the following inclusion criteria: adults (age over 18 years old at the time of recruitment), admitted with symptoms of impaired consciousness, including coma and akinetic mutism. Patients with diagnosed brain death, decerebration, and decortication were excluded from the study. The entire study was conducted in line with the rules and regulations of the institutionally approved research protocol. According to the General Data Protection Regulation the age of patients (in the day of conducting the research) has been presented in the form of ranges and the gender information has not been provided (Table 1).

Two experimental sessions were conducted with each patient. The second session was performed on the following day from the first one. The choice of tasks and the duration of tasks depended on the current patient state at the time of the experiment. The reactions and the performance of the patients were carefully observed by the therapist. All technical challenges with conducting the experiments with this patient population are summarized in Table 2.

**Table 2:** Challenges encountered during the diagnostic sessions.

	**Session 1**	**Session 2**
P1	Gaze fixation (poor patient cooperation and delayed responses)	Gaze fixation (poor patient cooperation and delayed responses)
P2	Gaze fixation (visual perception impairment)	Gaze fixation (visual perception impairment)
P3	–	–
P4	Gaze tracking (right eye utterly impaired; left eye ptosis)	–
P5	–	Gaze tracking (drowsy; no eye movement activity)
P6	Gaze fixation and saccade tracking	Gaze fixation and saccade tracking
P7	–	–
P8	–	–
P9	–	Gaze fixation (occasional technical issues; gaze shifts in direction of the correct answer)
P10	Gaze fixation (occasional technical issues; gaze shifts in direction of the correct answer)	Gaze fixation (occasional technical issues; gaze shifts in direction of the correct answer)

### Cyber Eye Hardware and Software

**Figure 1:**
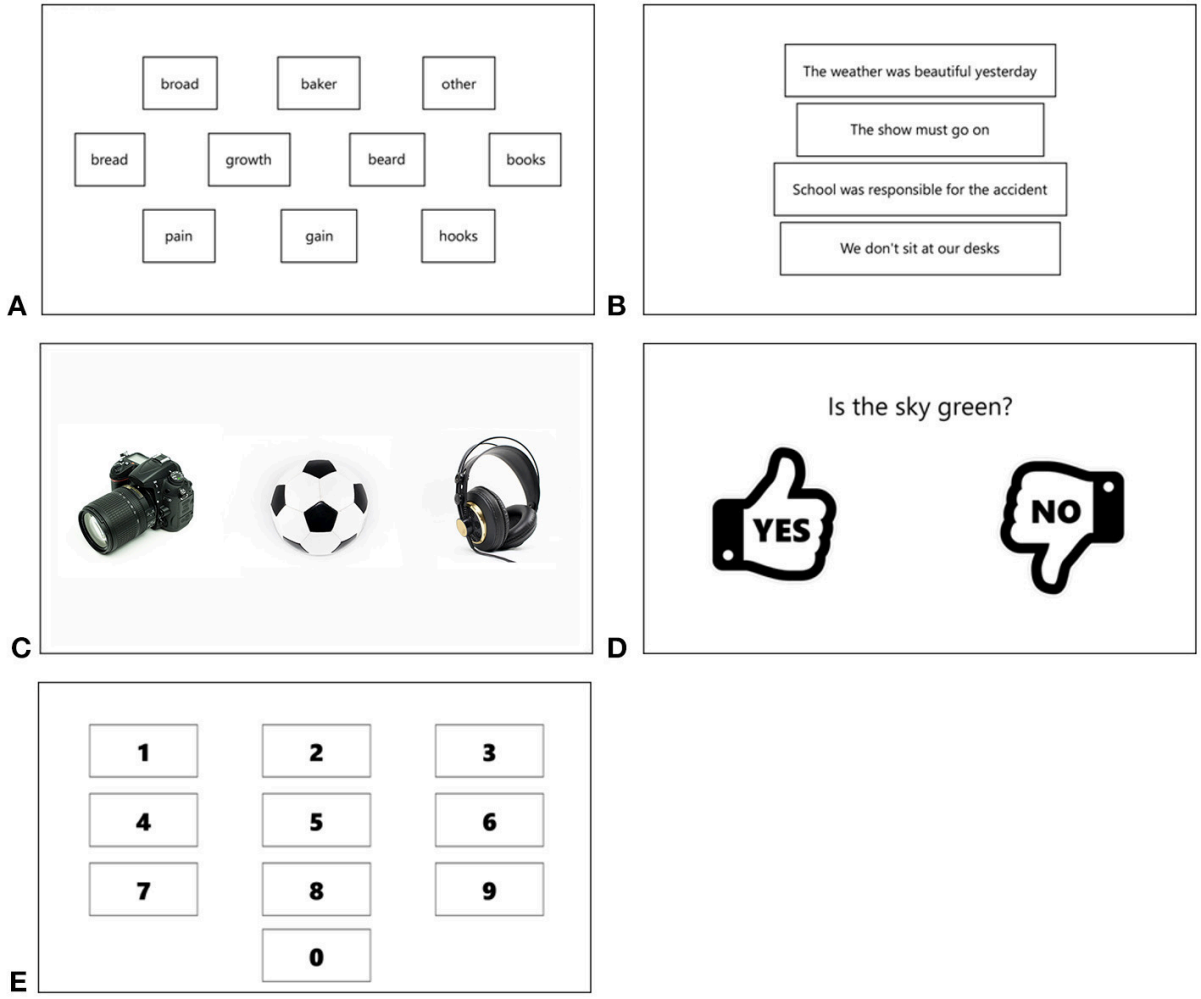
Sample views of the screen in tasks No. 1–5 **(A–E)**.

Our HCI consisted of “Tobii” eye gaze tracker hardware component (22), two monitor displays and speakers connected to a regular computer. We developed our own software with user-interface for research purposes. On one of the two monitors attached to the computer, the task content was presented to the patient. On another monitor, managing panels were displayed to the therapist. The whole solution was named “Cyber Eye,” which comprised five diagnostic tasks assessing cognitive functioning of a patient. For each task a few therapeutic sets were prepared. In Figure 1 views of a patient's screen in each task are presented. Below, a description of each task is provided.

*Task No. 1—“Indicate the heard word”* Various words are displayed on the screen (Figure 1A). The task is to indicate with gaze a word spoken by a speech synthesizer. When the patient indicates the word (either correctly or not) a new word is randomly chosen by the application and it is generated by the synthesizer.
*Task No. 2—“Indicate the heard sentence”* Four sentences are displayed on the screen, one under another (Figure 1B). The task is to indicate by gaze the sentence articulated by the speech synthesizer.
*Task No. 3—“Indicate the image”* Three images are displayed on the screen (Figure 1C). A word describing a content of the particular image is articulated by the speech synthesizer. The task is to indicate the image associated with the spoken word.
*Task No. 4—“Yes/No questions”* Pictograms representing positive and negative answers are displayed on two sides of the screen (Figure 1D). A question is articulated by the speech synthesizer. The task is to answer the question by looking at one of the pictograms.
*Task No. 5—“Indicate the digit”* Ten digits are displayed on the screen as presented in Figure 1E. The task is to indicate by gaze a digit articulated by the speech synthesizer. The digit is randomly selected by the application.

In tasks No. 1, 2, 3, and 5, when a patient indicated the ordered object (either correctly or not), a new object was randomly chosen by the application and its name was articulated by the synthesizer. A therapist could proceed to the next set of patterns using the managing panel. In each set related to the task No. 4 only one object (one question) was provided.

While the application was working, the gaze position and time stamps were registered in a log file for the purpose of further results analysis. Information about the start and the end of a particular task and about the chosen set were also contained in the log file. For each task the ordered object name and the name of the object selected by a patient were registered.

### Accuracy of Gaze-Tracking

**Figure 2:**
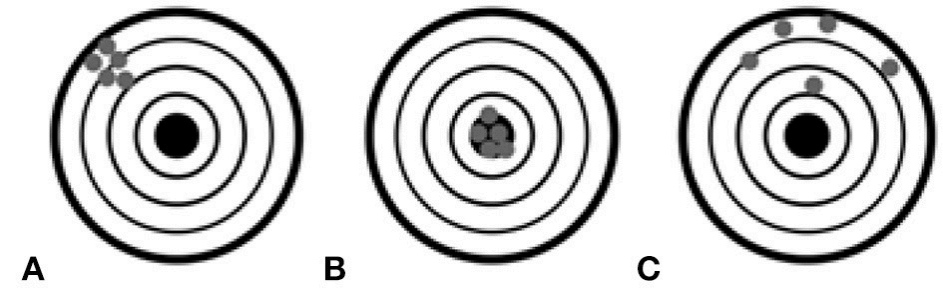
The accuracy and precision of the gaze tracker data recordings in three combinations: poor accuracy but good precision **(A)**, good accuracy and good precision **(B)**, and poor accuracy and poor precision **(C)** (23).

Two parameters are associated with quality of recorded gaze tracking data, i.e., accuracy and precision. Accuracy was defined as the average difference between the real stimuli position and the measured gaze position (23). Precision was defined as the ability of the eye tracker to reliably reproduce the same gaze point measurement. It can be represented as the variation of the recorded data via the Root Mean Square (RMS) of successive samples (23). Both parameters are graphically presented in Figure 2.

According to information from the Tobii gaze tracker website (23), “the accuracy error varies considerably across participants and experimental conditions. Accuracy is dependent on participant properties, illumination in the test environment, stimuli properties, calibration quality, data collection procedure and the eyes' position in the track box.”

The experimenter was blinded to the ability of the patients to perform the calibration process as it would have created potential biases about the consciousness state. Therefore, the calibration was performed by the therapist at the cost of accuracy and precision. While the application was working, a green dot was displayed on the screen in the location where the fixation point was detected. Without applying any averaging algorithm the dot trembled vastly as the fixation point coordinates oscillated rapidly. It caused a sight fatigue making the system harder to control, especially for people with brain and vision impairments. Therefore, the method of smoothing the fixation point based on the Kalman filter (24) was developed. Thus, the poor precision was compensated. The poor accuracy, on the other hand, leads to real gaze point offset. Patients with acquired brain injuries often have impaired visual perception (including saccade movement impairment) (25, 26). That can also affect convergence between the real gaze point and the detected one. Therefore, the method for compensating the gaze point offset was developed and it has been described beneath.

### Method for Compensating the Gaze Offset

**Figure 3:**
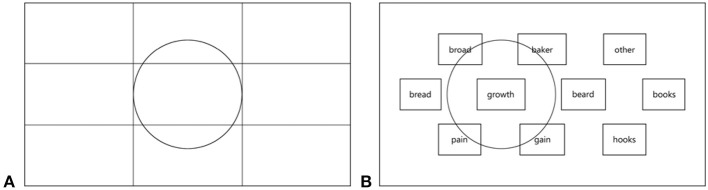
The way of calculating the gaze point offset compensation threshold in the tasks No. 1 and 5 **(A)** with the graphical representation of the threshold in task No. 1 **(B)**.

The method for compensating the gaze offset was designed during the process of developing the system. A screen image was divided into nine rectangles, as presented in Figure 3A. A circle was inscribed in one of the rectangles in such a way that its diameter was equal to the longer side of the rectangle. For each element embedded within the graphical user interface (GUI) in tasks No. 1 and 5, the center (*x*_*c*_, *y*_*c*_) point was designated. The element indicated to be selected by gaze was considered properly selected if the distance *d* between the point (*x*_*c*_, *y*_*c*_) and the gaze point (*x*_*g*_, *y*_*g*_) was smaller than the radius (threshold) of the above mentioned circle. The distance *d* was calculated using Euclidean metrics (Equation 1).

(1) $$\begin{array}{l} d=\sqrt{{\left(x_{g}-x_{c}\right)}^{2}+{\left(y_{g}-y_{c}\right)}^{2}} \end{array}$$

For screen resolution 1,920 × 1,080 and the above described methodology, the threshold was equal to 320. According to the graphical representation of the method (Figure 3B), the element was selected if the gaze point belonged to the area designated by the circle.

In task No. 2, each sentence occupied the substantial horizontal length of the screen, thus the Euclidean distance could not be used. Instead, the distance and the threshold were calculated only in *y*-axis, according to Equations (2, 3). Considering the placement of frames with sentences on the screen (Figure 1B) and considering height of the frames, the threshold was set to 150 pixels, by adopting constant value *k* = *a*/4, where *a* was the height of a sentence frame.

(2) $$\begin{array}{l} d^{\text{II}}=|y_{c}-y_{g}| \end{array}$$

(3) $$\begin{array}{l} d_{THR}^{\text{II}}=|y_{c}-y_{c+1}|-k \end{array}$$

**Figure 4:**
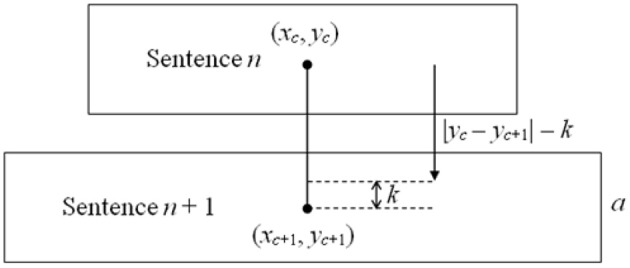
The way of calculating the distance threshold in the task No. 2.

The method of calculating the distance threshold in the task No. 2 is graphically presented in Figure 4. In tasks No. 3 and 4 no threshold was applied as the GUI elements were big enough to compensate the gaze fixation offset.

### Statistical Analysis

The correctness of choosing the GUI element was measured on a dichotomous scale with value 1 meaning the correct selection and value 0 meaning the wrong selection. For each task a vector was created which contained a distribution of answers reflecting the probability of choosing the proper GUI element. The vector is named random vector herein. For example, for the task No. 2, repeated 12 times, the vector adopted values {1, 0, 0, 0, 1, 0, 0, 0, 1, 0, 0, 0}, as each time four sentences were visible on the screen, and thus the probability of choosing the proper sentence was equal to 1/4. Therefore, every fourth answer in the vector was considered the proper one and it was denoted by “1.” In the task No. 1, 10 words were visible on the screen, thus the probability of choosing the proper answer was equal to 1/10. Therefore, the random vector contained one digit “1” per 10 occurrences. The vectors created in this way for every task were compared in one-tailed Fisher's exact tests with vectors containing answers given by the patients. This particular test was chosen for the following reasons: dichotomous scale utilized (1—correct, 0—wrong); in some cases the contingency table contained values not >5 (therefore, the chi-squared test could not be used). The distributions were asymmetric, i.e., the results in which the random vector contained significantly more proper answers than the patient's responses vector were to be treated as highly insignificant (meaning patient did not show symptoms of consciousness). Therefore, one-tailed tests were utilized. The significance level α was set to 0.05. In Table 3, a sample distribution of patient responses, illustrating the procedure in task No. 1, is given. As the distances between the gaze points over words *broad* and *baker* (Figure 1A), and the center of the indicated word *growth* had been smaller than the threshold = 320, these words were considered as proper indications. Thus, the correctness measure for them was equal to 1 (Table 3, bold). The *p*-value was equal to 0.015 and the odds ratio was equal to 23.496, meaning that the patient had about 23 times greater odds of being conscious than the patient responding with a random distribution.

**Table 3:** Example of patient responses in task No. 1.

**“Random” distribution**	**1**	**0**	**0**	**0**	**0**	**0**	**0**
Ordered word	Bread	Beard	Growth	Other	Books	**Broad**	**Baker**
Selected word	Bread	Broad	Growth	Other	Books	**Growth**	**Growth**
Correctness	1	0	1	1	1	1	1
Euclidean distance	160	648	183	54	46	277	291

## Results

Ten patients (four females) were involved in this observational study conducted in “EPIMIGREN” medical center in Osielsko, Poland. These 10 patients were chosen from the group of 14 patients who had been at that time under hospital care in this particular medical center. Based on the inclusion criteria, described in Material and Methods, one patient was excluded from this study based on the age criterion and the remaining three patients were excluded due to decerebration/decortication. We found that 6 out of 10 patients expressed conscious task performance in at least one of the five HCI tasks (Table 4; *p*-values for statistically significant results in bold). Meanwhile, one of them (P4) was originally (and successively) diagnosed as remaining in vegetative state (acute phase GCS score of 6), and one (P9) as being in coma (acute phase GCS score of 7) (Table 4, Figure 6). Ranking the patients in descending order by the median odds ratios (Fisher's exact test) resulted in identification of the patients P4 and P9 as performing with the second and third best result, respectively (Figure 6). The consciousness state of patient P4 enabled him/her to complete 3 tasks with performance higher than chance level (Figure 6 and Table 4; Fisher's exact test, *p* < 0.05, *n* > 5). This finding corroborated a suspicion of the therapist that the patient remained in the locked-in state, despite obtaining the second lowest GCS score among the 10 patients.

**Table 4:** Results of the Fisher's exact test (*p*-values) and the chronic neurological examination.

	**Diagnosis**	**Task 1**		**Task 2**		**Task 3**		**Task 4**		**Task 5**	
		**S1**	**S2**	**S1**	**S2**	**S1**	**S2**	**S1**	**S2**	**S1**	**S2**
P1	VS	0.786	1.0	0.720	0.786	0.5	0.787	0.738	0.843	0.763	0.500
P2	VS	0.152	1.0	0.089	0.121	0.152	1.0	0.681	0.988	–	–
P3	VS	1.0	0.285	0.500	0.500	0.621	0.265	0.767	0.500	0.500	0.500
P4	VS	**0.029**	0.291	0.500	0.333	0.795	0.675	0.500	**0.035**	0.500	**0.010**
P5	MCS	**0.015**	0.280	0.137	0.083	0.091	0.096	**0.015**	0.330	**0.016**	–
P6	AM	–	0.778	–	0.071	0.182	0.265	0.500	**0.039**	–	–
P7	MCS	0.893	0.893	0.664	0.132	0.893	0.889	0.904	0.658	0.500	**0.026**
P8	MCS	0.500	0.778	0.500	0.500	0.055	0.170	**0.038**	0.391	0.500	0.066
P9	coma	–	–	0.500	0.121	0.656	0.372	0.200	**0.014**	0.262	–
P10	VS	0.500	–	–	–	0.197	0.500	0.091	0.704	0.786	–

**Figure 5:**
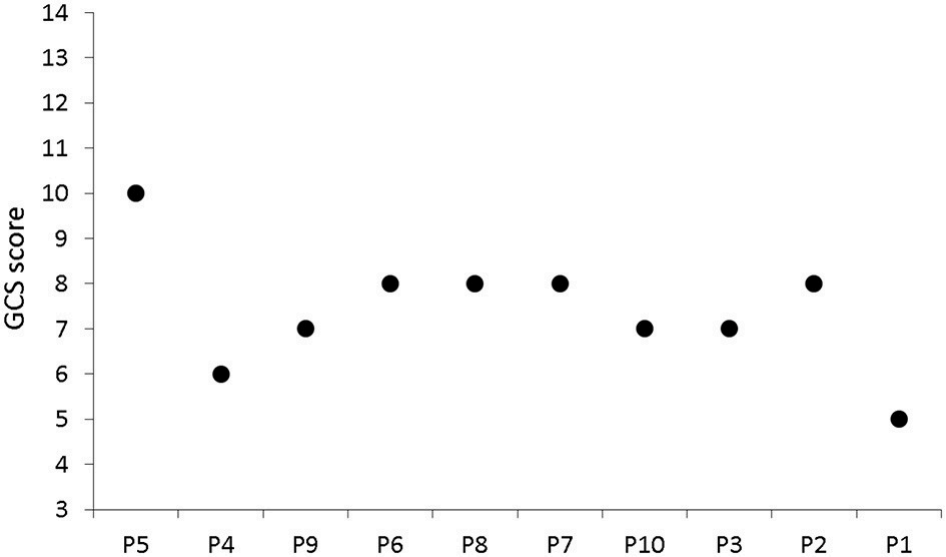
Results of GCS assessment in the acute phase.

**Figure 6:**
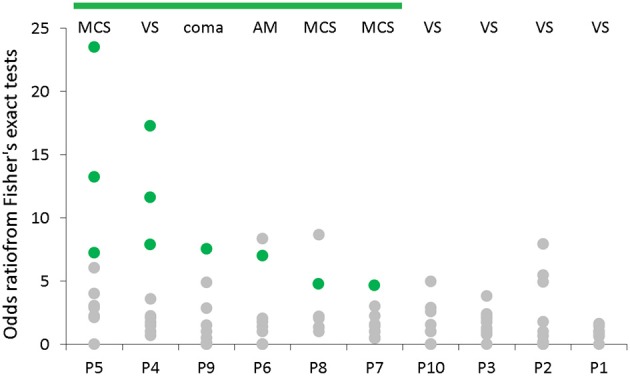
Comparison of the results of neurological examination with odds ratios-based scores obtained by employing the developed system. Abbreviations denoting the medical diagnosis are contained; for each patient the results from both sessions for each task are spread vertically; the patients are ranked in descending order by the median odds ratios; result of each session is presented as a green or a gray dot corresponding to a conscious (*p* < 0.05) and an unconscious (*p* ≥ 0.05) state, respectively. MCS, minimally consciousness state; VS, vegetative state; AM, akinetic mutism.

Despite a clear indication to use the GCS only in the acute phase of acquired brain injury, it is often practiced to score consciousness state chronically. In this scenario, comparing the results obtained using the developed HCI with the ones from the GCS assessment, would have revealed congruency for patients P5 and patient P1 only. According to the HCI obtained results, it appears that the acute phase GCS scores between 6 and 8 would not reflect the chronic state differences amongst patients. The most disparate diagnosis outcome in this critical range of the acute phase GCS scores occurred for patient P4, who obtained only 6 GCS points (Figure 5), was subsequently diagnosed as remaining in a vegetative state, and was re-diagnosed with a locked-in syndrome after applying our HCI. Another pertinent example of patient P9 obtained 7 GCS points acutely and was subsequently diagnosed as being in coma. Meanwhile, the patient performed one of the tasks and obtained odds ratio 7.52 higher than a randomly responding patient (Figure 6).

It is clinically accepted that a patient remaining in the chronic vegetative state scores 8 ± 1 GCS points in the initial acute phase, and a patient remaining in the minimally conscious state scores 11 ± 1 GCS points (27). Our results showed that 6 patients were conscious to some extent (Fisher's exact test, *p* < 0.05, *n* > 5), suggesting that the acute phase GCS assessment would generally underestimate the predicted chronic state. Interestingly, patient P7 who was diagnosed as being in the minimally conscious state scored only 8 GCS points acutely. The developed system showed that the patient was conscious enough to complete one of the tasks correctly, despite the lowest odds ratio-based score amongst the patients showing the symptoms of consciousness (Figure 6).

## Discussion

Employing eye-tracking technology into clinical practice of identifying the state of consciousness presents several challenges. In this study, in particular, patient performance depended not only on brain impairment kind and severity, but also on their mental and physical state during the diagnostic session. Moreover, as presented in Table 2, the patients suffered from other impairments like eye ptosis, severe eye movement impairment, visual perception impairment, and saccade movement impairment. In some cases, the state of a patient on a particular day made the HCI sessions impossible to conduct. For example, because of the impairment of a right eye and the left eye ptosis, patient P4 had problems with gaze fixation in tasks 2–5 during session 1 (Table 2). However, the patient was able to perform two tasks correctly (4 and 5) in session 2 despite these impairments. Similarly, problems with the gaze tracking observed for the patient 5 in session 2 prevented proper task performance, e.g., task 5 was not possible at all. Nonetheless, these problems were not observed in session 1, in which the patient was able to complete three tasks (1, 4, and 5). In general, a patient was identified as conscious if the tasks were completed with a significant score in either of the two sessions. Our Human Computer Interface may therefore require repeated sessions to overcome such limitations present on this challenging group of patients.

There are several aspects to be improved in the HCI system. Currently, the system does not detect deliberate, conscious, wrong selections made by a patient while performing the tasks. In this hypothetical case, the assessment using our HCI would show lack of consciousness. In comparison, the risk of this error of omission is minimal while using scales testing for reaction to pain (e.g., Coma/Near Coma Scale) as refraining from reacting to painful stimuli is difficult. Another limitation of the HCI system is associated with relying on the mental and physical state of patients, which is highly variable. In general, the clinical scales with qualitative neurological assessment can be used with non-cooperative patient, whereas the HCI-based sessions require a patient to be in a relatively good condition on a particular day and to be willing to complete the tasks. Thus, our HCI could not be utilized in the acute phase of injury. Still, the developed HCI approach is proposed as a new complementary tool rather than a replacement for the clinical assessment of patients suffering from disorders of consciousness. Instead of classifying definite states of a patient, HCI ranks their scores based on the odds ratios to provide more precise information and thus improve identification of the actual state of consciousness. Nonetheless, differentiation between a vegetative state and coma would be still performed using standard neurological assessment and various scale-based methods other than our HCI. Similarly, distinguishing a minimally conscious state from akinetic mutism cannot be performed based exclusively on the odds ratios. However, our HCI system could be particularly useful in assessing the level of consciousness impairment in conscious patients and in identifying the locked-in syndrome.

The software development kit (SDK) provided with the Tobii gaze tracking system offered a method of filtering the raw gaze position signal. The method, however, resulted in poor precision, unstable and noisy measurements, intensifying the mouse chase effect (28, 29). Therefore, we developed a method of fixation point smoothing based on the Kalman filter and utilized it instead of the method provided with the SDK. This enabled us to create an application controllable even by patients with vision impairments. However, the level of smoothing of the fixation point depended on the parameter set arbitrarily based on the performance of the particular processing unit and frame rate of the gaze tracker. When combining the described procedure with another hardware, one should consider adjustment of this parameter in order to obtain the best possible smoothing of the gaze position. Choosing an arbitrary value of the parameter could potentially affect assessment of the actual level of consciousness.

Our aim was to present the tool complementary to the scales used clinically. We wanted to avoid creating an impression that our new HCI is an alternative option to replace the existing scales, e.g., The Four Scale (13). Our point is rather that HCI can be a tool complementary to the clinically used scales to improve their accuracy with more quantitative measures. For this reason, and due to the fact that it is not recommended to use the GCS with patients in the chronic phase, we do not directly compare the results of the GCS assessment with the results of the assessment with our HCI method. Besides, these cannot be compared statistically as the magnitudes of the GSC are qualitatively incompatible with the odds-ratio measure. The GCS has a fixed scale with a minimum and maximum values whereas no maximum value can be set for the odds ratios in our approach. High odds ratio values do not determine the significance effect unless *p*-value is smaller than the significance threshold (*p* < 0.05 in our study). Hence, the only way to compare the data statistically would be to classify the results as conscious/unconscious and to check if the differences in number of labels among groups is significant. The Fisher's exact test could be used for such a purpose as the scale would be dichotomous. However, such statistics might still be considered inappropriate and would create an impression of replacing the GCS, which was not the goal of this work, and which should not be practiced beyond the acute phase. In conclusion, our HCI system provides an alternative approach to assess the state of consciousness, which can complement the standard clinical methods. We found that the HCI contributed to improved diagnosis accuracy as compared to the currently practiced neurological examinations. The results obtained using the system exposed in another way the shortages of acute phase GCS assessment, reported earlier in the literature (2, 4, 30–32), and the need for complementary quantitative methods for chronically used neurological scales. The emerging technologies for communication between the human brain and the computer will continue to supplement and transform medical practice.

## Data Availability Statement

The raw data supporting the conclusions of this manuscript will be made available by the authors, without undue reservation, to any qualified researcher.

## Ethics Statement

Research was conducted on the basis of 2 Ethics Committee approvals of the Collegium Medicum Ludwika Rydygiera (part of Nicolaus Copernicus University), 85-067 Bydgoszcz, Poland.

1. decision No. KB 115/2017 issued on February 2, 2017
2. decision No. KB 56/2018 issued on February 27, 2018

## Author Contributions

ML developed the software, collected and analyzed the data, and wrote the manuscript. MK edited the manuscript and contributed to data analysis and presentation. AC designed the study and edited the manuscript.

### Conflict of Interest Statement

The authors declare that the research was conducted in the absence of any commercial or financial relationships that could be construed as a potential conflict of interest. The software for research purposes in clinical application was developed by the authors and is not available commercially. Commercially available Tobii gaze tracker was utilized in the system hardware.
